# Fluralaner injectable suspension (Bravecto® injectable) for dogs remains effective against *Ixodes ricinus* infestations for one-year

**DOI:** 10.1186/s13071-025-06963-0

**Published:** 2025-08-09

**Authors:** Georg von Samson-Himmelstjerna, Christina S. Helm, Jürgen Krücken, Eva Zschiesche, Lea Heinau, Ard M. Nijhof, Ivo Petersen

**Affiliations:** 1 Institute for Parasitology and Tropical Veterinary Medicine, Freie Universität Berlin, Berlin, Germany; 2 Veterinary Centre for Resistance Research (TZR), Freie Universität Berlin, Berlin, Germany; 3 MSD Animal Health Innovation GmbH, Zur Propstei, Schwabenheim an der Selz, Germany

**Keywords:** Fluralaner, *Ixodes ricinus*, Dog

## Abstract

**Background:**

Tick-borne pathogens are emerging in many regions worldwide with seasonal transmission often shifting to year-round transmission in temperate climatic zones due to climate change. This situation makes year-round protection against ticks advisable. The objective of the study was to confirm the efficacy of a single injection of fluralaner against *Ixodes ricinus* ticks for up to 56 weeks.

**Methods:**

The study was a single-site, blinded, negative controlled, randomized efficacy study conducted with 20 female dogs. Dogs were randomized into two groups based on tick counts at pre-infestation. Dogs were infested with approximately 45 *I. ricinus* female ticks plus 5 male ticks at 17 time points over a year. Bravecto^®^ injectable (15 mg fluralaner /kg BW) as Investigational Veterinary Product (IVP) was administered once as a subcutaneous injection 48 h after the first experimental infestation. Female ticks were counted at 48 ± 4 h post-treatment or post-infestation to evaluate immediate and persistent efficacy, respectively.

**Results:**

The tick infestation per infestation day recorded from individual dogs in the negative control group ranged from 0 to 58. At each counting time point, at least seven dogs in the negative control group were infested with ≥ 25% of the ticks placed on them, indicating an adequate tick infestation and a sufficient tick challenge at each time point. The IVP was well tolerated in all dogs. Significantly fewer (p < 0.0001) live attached ticks were recorded from the IVP treated group compared to the control group at each time point. Based on arithmetic means of live attached tick counts, the IVP was 94.97% effective 7 days after administration. Persistent efficacy over one year following IVP administration was confirmed. The treatment efficacy after 12 and 13 months was 99.16% and 96.22%, respectively.

**Conclusions:**

Based on the significant reduction in mean live attached *I. ricinus* counts compared to the negative control group, efficacy based on arithmetic mean ≥ 90%, and adequacy of infestation in the control dogs, the IVP was effective against existing and new *I. ricinus* tick infestations for up to 394 days following administration.

**Graphical Abstract:**

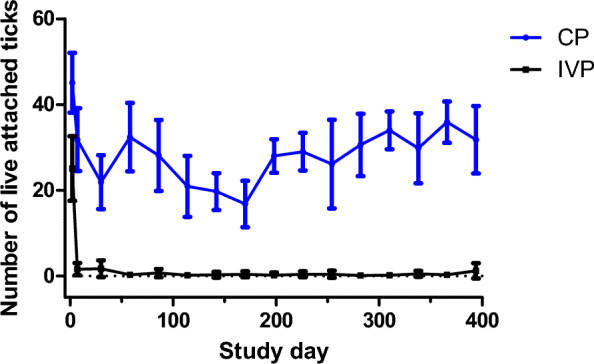

## Background

Like other animals with access to the environment, dogs are regularly exposed to tick infestations. In Germany, as in other countries in Western Europe, the most prevalent and abundant species found on dogs are the so-called castor bean tick, *Ixodes ricinus,* and the meadow tick, *Dermacentor reticulatus* [1, 2]. *Ixodes ricinus* is known to be distributed throughout Germany with no major differences concerning its geographical spread [3, 4]. During the course of the year, *I. ricinus* mostly exhibits a bimodal seasonal activity pattern with activity peaks in spring and autumn [5, 6]. However, under the prevailing climatic conditions of recent years, it has also been shown, that *I. ricinus* may exhibit a year-round activity pattern [2, 7].

In most cases tick infestation does not result in clinical disease. However, in some animals it can lead to clinical signs including local skin reaction, pruritus, alopecia and, depending on the intensity of the tick burden as well as the age of the host, also to more complex diseases, e.g., due to bacterial skin infections or even severe symptoms such as anemia. Considerably more relevant than the direct consequences of tick bites are those of tick-transmitted diseases. For *I. ricinus* and dogs, these are mainly bacterial pathogens such as *Borrelia* spp. and *Anaplasma phagocytophilum*. In a recent study of 1500 female ticks removed from dogs during a nationwide geographic distribution study in Germany, the prevalence for *Borrelia* spp. was 28.5% and that of *A. phagocytophilum* 19.0% with 6.9% co-infections [8]. *Borrelia burgdorferi* sensu stricto, which is associated with severe clinical cases in dogs [9], has a regionally varying prevalence in Germany. In a study in southern Germany, the following prevalences were observed for the identified *Borrelia* species: *B. afzelii* 11.7%, *B. burgdorferi* s.s. 3.7%, *B. garinii* 3.2%, *B. valaisiana* 1.8%, *B. spielmanii* 1.5% and *B. bavariensis* 0.2% [10]. Furthermore, tick-borne encephalitis virus (TBE) is transmitted by *I. ricinus* and has been described to lead to severe, sometimes even fatal, disease in dogs [11, 12].

To treat dogs infested with ticks, and to prevent the transmission of pathogens, a broad spectrum of options is available. Through regular visual inspection of all body parts with appropriate scrutiny (i.e., parting of the hair to ensure complete sight of the skin) ticks can be found and then removed manually using appropriate instruments (e.g., tick tweezers). However, with this approach there is a considerable risk of overlooking ticks, particularly in larger dogs and those with long fur [13, 14]. There are several ectoparasitic drug classes available for tick control, such as the pyrethroids, carbamates, phenyl pyrazoles and isoxazolines [15]. The latter is the newest acaricidal drug class with various compounds including fluralaner developed as both oral and spot-on products with or without additional endoparasiticides [16–19]. Within the isoxazoline substance class, fluralaner containing veterinary medicinal product-formulations exhibit long-term acaricidal efficacy of at least 8 to 12 weeks, depending on the dosage form and target tick species [20–22]. In comparison, products containing other acaricidal compounds including other isoxazoline substances, are only effective for around four weeks [23–27].

To effectively prevent tick infestation, and thus also the transmission of tick-borne pathogens, continuous administration of acaricidal products is recommended by expert organizations such as the European Counsel for Companion Animal Parasites (ESCCAP) [28] or the US based Companion Animal Parasite Council (CAPC) [29]. Consequently, given the year-round activity of *I. ricinus* as well as *D. reticulatus* in Western Europe, there is a need for protection during the entire year. Recently, an innovative pharmaceutical formulation of fluralaner in the form of an injectable suspension has been registered in several regions world-wide. This product has been shown to provide 12 months protection against *Rhipicephalus sanguineus, Dermacentor spp.* and *Ixodes spp.* ticks as well as *Ctenocephalides spp.* fleas [30, 31]. In the here described study, the safety and efficacy of a single administration of fluralaner injectable suspension at a dose rate of 15 mg/kg (0.1 mL/kg) body weight with monthly *I. ricinus* infestations in dogs over a period of 13 months was investigated.

## Methods

The study was conducted at the Freie Universität Berlin, Germany, at the Institute for Parasitology and Tropical Veterinary Medicine 2021 until 2022. The objective was to confirm the efficacy of fluralaner injectable suspension against *I. ricinus* for one year after a single administration at a dose of 15 mg/kg body weight. All procedures were in alignment with the principles of Good Clinical Practice VICH GL9 (GCP) [32] and according to the Guideline for Efficacy Testing of Antiparasitic Substances in Dogs and Cats by the European Medicines Agency (EMA) [33].

### Animals

The study animals were purpose bred female, intact Beagle dogs approximately three years of age at the start of the study. Each animal was individually identified by name and by using the last six digits of the nine digits of a subcutaneously implanted microchip. The animals received an age-appropriate commercial dog diet. All dogs were acclimatized and trained during the study period prior to infestation and treatment. From the day of randomization, the animals in one group had no contact with the animals in the other group. Outside of the tick infestation periods, the dogs were kept in groups in indoor kennels with outdoor runs. The staff wore protective clothing when caring for the animals and the equipment was separated for the two groups. From the time point of infestation until the tick count, the animals were housed individually in kennels.

The keeping of dogs complied with the requirements of Directive 2010/63/EU of the European Parliament and of the Council on the protection of animals used for scientific purposes [32].

### Study design

The study was a single-site, blinded, negative controlled, randomized efficacy study conducted with 20 dogs experimentally infested with *I. ricinus*. The study started with an initial *I. ricinus* tick infestation on study day (SD) −7 to evaluate the susceptibility of each dog to experimental infestation and for random allocation to the study groups. All suitable dogs were ranked by descending order of live attached tick counts. Dogs with the same live attached tick counts were ranked in ascending alpha-numeric order by dog identification number. Subsequently, dogs were blocked into 10 blocks of two dogs each. Within each block, dogs were randomly allocated to the two treatment groups (A and B). The randomization was performed using a computer-generated randomization list provided by the study statistician.

Over the entire duration of the study, all dogs were observed once daily for general health by a trained animal attendant. Each dog was noted as being normal or abnormal on the Daily General Health Observations form. Furthermore, each dog was physically examined by a veterinarian on SD -7 for enrollment and to ensure good health prior to the first tick infestation on SD -2. On SD 0, the General Health Observation was done once prior to treatment administration as well as thereafter monthly until the end of the study. All abnormal health observations observed after SD 0 until the last day of the study were also recorded. Additionally, all dogs were weighed for dose rate calculation for sedatives to be used for tick infestations and for exact dosing of the IVP or control product (CP).

### Tick infestation and count

The isolate was a European laboratory-bred strain of *I. ricinus* (Institute for Parasitology and Tropical Medicine, Freie Universität Berlin, Germany). The isolate was last enriched with field-collected ticks in 2020, i.e. the year immediately prior to the start of the study.

At each infestation, around 50 (± 4) viable unfed adult *I. ricinus* ticks, approximately 45 females and 5 males, were used and directly applied to the fur of each dog. For animal welfare reasons and to provide the ticks sufficient time to crawl into the hair coat, the dogs were sedated for tick infestations. For this purpose, the dogs were administered with 0.4 mg/kg butophanol and 0.02 mg/kg medetomidine via a single intravenous injection. Dogs were infested on SD -7, for randomization purposes, and then again on SD -2, to assess immediate efficacy, as well as on SDs 5, 28, 56, 84, 112, 140, 168, 196, 224, 252, 280, 308, 336, 364 and 392 to assess persistent efficacy.

Tick removal was conducted on each animal on SD −5, 7, 30, 58, 86, 114, 142, 170, 198, 226, 254, 282, 310, 338, 366 and 394, which was 48 h ± 4 h post infestation. On SD 2 ticks were counted and removed 96 h ± 4 h post infestation, respectively 48 h ± 4 h post treatment. At least two persons were involved in the tick count procedure. The dog was examined for ticks by pushing the hair manually against its natural hairline to expose the skin and any ticks that were present were removed gently using tick removal devices, forceps or fingers.

Live attached ticks and dead ticks from each dog were categorized according to attachment status (free or attached) immediately after removal by visual assessment. To determine whether the ticks were dead or alive, they were placed on a light-coloured, smooth surface to make it easier to see any movements. If necessary, they were stimulated by touch or examined under a stereomicroscope.

### Treatment

On SD 0, all 20 dogs were treated. There was one treatment group (fluralaner 150 mg/mL injectable suspension (Bravecto^®^ injectable ad us. vet.), IVP) and one negative control group (0.9% sterile saline solution, Control Product, CP). The dose to be administered was 15 mg fluralaner / kg BW. The injection was administered subcutaneously in the dorso-scapular region. Each dog was observed for injection site reactions on SD 0 at 10 min up to 1 h post treatment, on SD 1 at 24 h ± 60 min post-treatment, and on SD 2 (prior to tick count and removal), SDs 3, 4, 7, 10 and 14 ± 60 min post-treatment. If injection sites reactions were observed, they were monitored daily until resolution. Evaluations were continued daily until resolved. The injection site was observed and gently palpated for erythema, heat, pain, and swelling. Palpable swelling was qualified in its character, i.e., hard or soft. At each assessment erythema, heat, and pain parameter was scored as: 0 = no reaction. 1 = slight reaction. 2 = moderate reaction or 3 = severe reaction. Swelling was scored as none, palpable, or visible or palpable. The injection site was observed and gently palpated for erythema, heat, pain and swelling at 10 min up to 1 h post treatment and subsequently once per day around the same time treatment was done on SDs 1, 2, 3, 4, 7, 10 and 14.

An Adverse Event (AE) was any observation in study animals that occurred after the use of the IVP, whether or not considered to be product related, that was unfavourable and unintended. For blinding reasons, AEs occurring in the control group were also notified, documented, and reported.

### Statistical analysis

The statistical analysis was performed by means of the software package SAS®, release 9.4 (SAS Institute Inc., Cary, NC, USA).

Each dog represented an experimental and statistical unit. The Full-Analysis-Set (FAS) population comprised all animals that were enrolled in the study. The Per-protocol (PP) population comprised all animals that were treated and examined according to the protocol. For animals that were excluded, only data until the time point of exclusion were kept in the PP population.

According to European Guideline on Statistical Principles for Veterinary Clinical Trials [34], as well as the guideline of the World Association for the Advancement of Veterinary Parasitology (WAAVP) [35], at least six dogs per group are recommended. Herein, a sample size of 10 dogs per study group was used. Acknowledging the risk of dogs being removed from the study during the more than one year-long study period, this number of dogs per group was considered adequate for testing the efficacy of the selected IVP. Initial homogeneity of study groups was evaluated descriptively in both FAS and PP population. Means, standard deviations, medians, minima and maxima were calculated to characterize the distribution of body weight [kg] on SD -2 and number of ticks on SD -5.

Tick efficacy evaluation was considered valid if at a certain assessment point at least 13 ticks (at least 25% of the infestation dose of ticks) were counted on at least six control animals at this assessment time point (5). Primary efficacy was evaluated in the PP population only. The primary efficacy criterion is percentage of tick efficacy in the IVP group (group B) in relation to the CP group (group A) at each assessment time point. The acaricidal efficacy required to obtain the efficacy claim is at least 90% at each tick infestation during the claimed efficacy period [33].

Efficacy was calculated using Abbott’s formula. The distribution of ticks per study group was summarized descriptively. The validity of the efficacy results was confirmed by a statistical comparison of the live attached tick counts in the IVP group to the live attached tick counts in the CP group using a linear mixed model including the study group as a fixed effect and the randomization block as a random effect. The level of significance for the fixed effect was set to α = 0.05 (two-sided).

Efficacy results based on geometric means were provided additionally. The assessment of safety was carried out for all dogs in the FAS population.

## Results

### Study dogs and tick infestation

A total of 20 dogs were included into the study (FAS) population. Ten dogs were treated with IVP and ten dogs were treated with the control product (CP). At the start of the study (SD -2) the dogs weighed between 7.8 and 11.3 kg with mean body weights of 8.7 (standard deviation, StD ± 0.5) and 9.1 (StD ± 1.1) kg in the IVP and CP group, respectively, with no notable differences between the study groups.

One dog was withdrawn from the study due to an ongoing, recurrent disease on SD 369. The dog was not excluded from the statistical analysis, but no efficacy data were available for this animal after SD 369. All other dogs completed the study according to the study protocol. Thus, FAS and PP population were identical and comprised 20 dogs. Mean tick infestations in the IVP and CP groups obtained on SD -5 were 30.7 (± 9.2) and 31.1 (± 5.3), respectively.

Throughout the course of the study, adequate tick infestations were obtained in at least eight animals in the CP group, with 12 infestations resulting in adequate infestation in all CP group animals (Table 1). Throughout the study, with the exception of one CP group animal at SD 254, all infestations led to a positive tick count and at least eight and up to 58 live attached ticks at 48 h post infection. In the CP group the mean tick count for all infestations was 28.4 attached live ticks (StD 9.2).

**Table 1:** Adequacy of tick infestation in the control product group (number of dogs infested with ≥ 13 ticks)

Study day	Tick infestation with *I. ricinus*	Study day	Tick infestation with *I. ricinus*
2	Yes (10 of 10)	198	Yes (10 of 10)
7	Yes (10 of 10)	226	Yes (10 of 10)
30	Yes (10 of 10)	254	Yes (9 of 10)
58	Yes (10 of 10)	282	Yes (10 of 10)
86	Yes (10 of 10)	310	Yes (10 of 10)
114	Yes (8 of 10)	338	Yes (10 of 10)
142	Yes (10 of 10)	366	Yes (10 of 10)
170	Yes (8 of 10)	394	Yes (8 of 9)

### Acaricidal efficacy

**Fig. 1:**
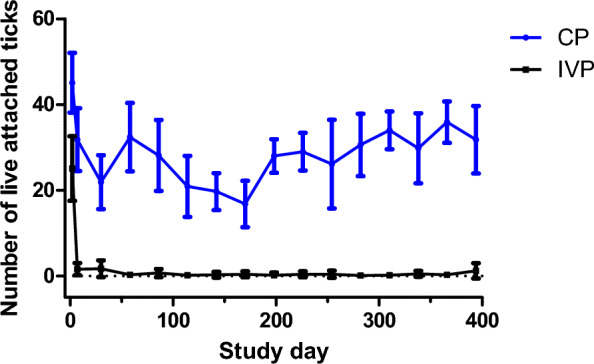
Course of the number of live attached ticks (arithmetic mean ± StD of ten dogs for each group) for the Investigational Veterinary Product (IVP) and Control Product (CP) groups during the entire study period

The acaricidal efficacy of the fluralaner suspension for each of the 16 infestations is summarized in Table 2. Starting from SD 7 onwards and until the study ended at SD 396 the efficacy, i.e., the reduction of (arithmetic) mean live attached tick counts was always above 92.2% while the difference in tick counts between the two study groups was always statistically significant with P-values always less than 0.0001. The mean tick infestations in the CP group did not show any major fluctuations during the course and also no declining trend at the end of the study (Fig. 1).

**Table 2:** Mean live attached tick counts (AM) efficacy and results of the statistical group comparison using a linear mixed model with study group as fixed and randomization block as random effect

Study day	Mean tick count CP group (AM)	Mean tick count IVP group (AM)	Efficacy [%]	Test statistic^a^	P value
2	45.10	25.10	44.35	F1.17 = 38.06	(p < 0.0001)
7	31.80	1.60	94.97	F1.17 = 164.83	(p < 0.0001)
30	21.90	1.70	92.24	F1.17 = 94.41	(p < 0.0001)
58	32.40	0.30	99.07	F1.17 = 161.42	(p < 0.0001)
86	28.10	0.70	97.51	F1.17 = 108.14	(p < 0.0001)
114	20.90	0.20	99.04	F1.17 = 84.48	(p < 0.0001)
142	19.70	0.30	98.48	F1.17 = 199.02	(p < 0.0001)
170	16.80	0.40	97.62	F1.17 = 89.65	(p < 0.0001)
198	28.00	0.20	99.29	F1.17 = 496.18	(p < 0.0001)
226	29.00	0.40	98.62	F1.17 = 567.35	(p < 0.0001)
254	26.10	0.40	98.47	F1.17 = 61.20	(p < 0.0001)
282	30.60	0.10	99.67	F1.17 = 216.03	(p < 0.0001)
310	34.00	0.20	99.41	F1.17 = 749.32	(p < 0.0001)
338	29.80	0.50	98.32	F1.17 = 127.06	(p < 0.0001)
366	35.90	0.30	99.16	F1.17 = 530.52	(p < 0.0001)
394	31.78	1.20	96.22	F1.16 = 158.90	(p < 0.0001)

^a^F statistics and degrees of freedom

AM, arithmetic mean

When calculating the geometric mean number of live attached ticks, the resulting efficacy was also above 94.9% from SD 7 until the end of the study (Table 3). Except for three study days at the beginning and end of the study, respectively, no more than two live attached ticks were encountered per dog in the IVP group.

**Table 3:** Geometric mean (GM), standard deviation (Std Dev), minimum (Min), median and maximun (Max) numbers of live attached tick counts and resulting efficacy

Study day	CP					IVP					
	Mean	Std Dev	Min	Median	Max	Mean	Std Dev	Min	Median	Max	Efficacy [%]
2	45.1	6.97	34	45	58	25.1	7.52	15	23	38	45.95
7	31.8	7.3	16	32	40	1.6	1.43	0	2	3	96.22
30	21.9	6.28	13	21	35	1.7	1.95	0	1	5	94.94
58	32.4	7.97	19	34	41	0.3	0.48	0	0	1	99.26
86	28.1	8.29	15	31	38	0.7	0.95	0	0	2	98.18
114	20.9	7.11	9	22	29	0.2	0.42	0	0	1	99.24
142	19.7	4.3	14	19	28	0.3	0.67	0	0	2	98.98
170	16.8	5.43	8	17	27	0.4	0.7	0	0	2	98.24
198	28	3.94	20	29	34	0.2	0.63	0	0	2	99.58
226	29	4.4	22	29	38	0.4	0.7	0	0	2	99.02
254	26.1	10.35	0	28	38	0.4	0.84	0	0	2	98.78
282	30.6	7.28	20	33	40	0.1	0.32	0	0	1	99.76
310	34	4.42	29	33	41	0.2	0.42	0	0	1	99.56
338	29.8	8.19	16	32	42	0.5	0.71	0	0	2	98.70
366	35.9	4.86	27	37	44	0.3	0.48	0	0	1	99.35
394	31.78	7.89	12	33	39	1.2	1.81	0	1	5	97.60

### Assessment of safety and adverse events

No abnormalities were observed in the study animals before or at inclusion. Following treatment, a slight erythema was observed at the injection site at 10 min post-injection of the IVP in one dog. This erythema was resolved at the follow-up inspection one hour post-injection. Other adverse events observed in the IVP group during the 13-month study duration included lameness, otitis externa and watery feces. These were always considered non-serious and they all resolved. None of the adverse events were considered to be associated with the IVP treatment, all lameness cases were caused by trauma (e.g., a dog bite wound).

## Discussion

Protection from tick infestation is a key requirement for all dogs not only due to the direct effects of tick bites but particularly as a consequence of highly prevalent tick-transmitted pathogens and the associated health risks. Given the ubiquitous distribution and year round activity of *I. ricinus*, a persistent and highly effective year-round tick protection is needed to prevent tick-associated diseases in dogs. Based on ectoparasiticide sales data from the US, this is not achieved in most dogs and for considerable time periods (ca. six months per year) about 50% of dogs have not been protected [36–38]. Similar challenges have been observed in Europe, where sales analyses suggest that a significant proportion of dogs are not continuously protected throughout the year, despite the widespread presence and long seasonal activity of ticks such as *I. ricinus* [39]. The period during which the dog will not be protected also depends on owner compliance and the duration of activity of the respective ectoparasiticide, and oral or dermal fluralaner products were demonstrated as providing longer coverage compared with other isoxazolines [40]. Nevertheless, even 80% of the dogs treated with fluralaner had a period of at least six months without protection [37].

One potential reason for non-compliance with the tick-protection recommendations provided by expert organizations such as ESCCAP or CAPC might be the requirement for frequent treatments. In human patients, studies concerning compliance in repeated daily medications showed that there was an association with better adherence to treatment regimens and the use of drugs with longer duration of activity [40, 41].

The fluralaner injectable suspension that was employed in the present study has previously been shown to be highly effective over 12 and 13 months against experimental infestations with *R. sanguineus* and *I. holocyclus* ticks, respectively [30, 31]. In these studies, the product was also found to be safe, with only one dog of the 30 treated dogs showing erythema at the injection site which resolved without treatment within 24 h [30]. In the present study the acaricidal activity against the castor bean tick *I. ricinus* also lasted over 13 months, with more than 96% efficacy observed at one year post treatment (for both calculations using AM and GM, respectively). The treatment was safe with only one minor and quickly resolving episode of injection-site erythema. In this study no other parasiticide, and except analgesics, antibiotic eye drops, antibiotic ear drops and sedatives, no other medications were administrated to the study dogs. Accordingly, the fluralaner injectable suspension provides an innovative option for *I. ricinus* control, promising longer annual protection periods and thus better protection from tick transmitted diseases. In this context it is also relevant to consider the time required for killing attached ticks, as this has a major influence on the risk of pathogen transmission. For infections spread by *I. ricinus*, the published transmission times vary depending on the pathogen. *B. burgdorferi* s. s. typically requires 24 to 48 h of tick attachment for efficient transmission [42, 43], though early transmission (within 17 h) has occasionally been reported [44]. In contrast, *A. phagocytophilum* can be transmitted more rapidly, with experimental studies demonstrating transmission as early as 24 h, and in some cases even under 16–18 h post-attachment [45]. These timeframes highlight the importance of fast-acting acaricides to minimize the risk of infection.

## Conclusions

This study showed that successful repeated monthly infestations of dogs with adult *I. ricinus* ticks over a period of over one year to test the efficacy of acaricidal drugs is feasible. Based on the significant reduction (p ≤ 0.01) in mean live attached *I. ricinus* counts compared to the control group, efficacy based on arithmetic mean ≥ 90% and adequacy of infestation in the control dogs, the IVP (Fluralaner 150 mg/mL injectable suspension) was effective against existing and new *I. ricinus* infestations for one year following administration. The IVP was also generally well tolerated in dogs.

## Data Availability

Data supporting the main conclusions of this study are included in the manuscript.

## References

[1] Beck S, Schreiber C, Schein E, Krücken J, Baldermann C, Pachnicke S, et al. Tick infestation and prophylaxis of dogs in northeastern Germany: a prospective study. Ticks Tick Borne Dis. 2014;5:336–42. 24629616 10.1016/j.ttbdis.2013.12.009

[2] Probst J, Springer A, Strube C. Year-round tick exposure of dogs and cats in Germany and Austria: results from a tick collection study. Parasit Vectors. 2023;16:70. 36797779 10.1186/s13071-023-05693-5 PMC9933410

[3] Rubel F, Brugger K, Monazahian M, Habedank B, Dautel H, Leverenz S, et al. The first German map of georeferenced ixodid tick locations. Parasit Vectors. 2014;7:477. 25301245 10.1186/s13071-014-0477-7 PMC4196197

[4] Rubel F, Brugger K, Chitimia-Dobler L, Dautel H, Meyer-Kayser E, Kahl O. Atlas of ticks (Acari: *Argasidae, Ixodidae*) in Germany. Exp Appl Acarol. 2021;84:183–214. 33939100 10.1007/s10493-021-00619-1 PMC8102463

[5] Schulz M, Mahling M, Pfister K. Abundance and seasonal activity of questing *Ixodes ricinus* ticks in their natural habitats in southern Germany in 2011. J Vector Ecol. 2014;39:56–65. 24820556 10.1111/j.1948-7134.2014.12070.x

[6] Pfäffle M, Petney T, Skuballa J, Taraschewski H. Comparative population dynamics of a generalist (*Ixodes ricinus*) and specialist tick (*I. hexagonus*) species from European hedgehogs. Exp Appl Acarol. 2011;54:151–64. 21350974 10.1007/s10493-011-9432-x

[7] Probst J, Springer A, Topp AK, Bröker M, Williams H, Dautel H, et al. Winter activity of questing ticks (*Ixodes ricinus* and *Dermacentor reticulatus*) in Germany - evidence from quasi-natural tick plots, field studies and a tick submission study. Ticks Tick Borne Dis. 2023;14:102225. 37399628 10.1016/j.ttbdis.2023.102225

[8] Probst J, Springer A, Fingerle V, Strube C. Frequency of *Anaplasma phagocytophilum*, *Borrelia *spp., and coinfections in *Ixodes ricinus *ticks collected from dogs and cats in Germany. Parasit Vectors. 2024;17:87. 38395915 10.1186/s13071-024-06193-w PMC10893606

[9] Hovius KE, van den Bergen T, Almalik O, Versmissen E, Rutten VP, Sprong H, et al. Clinical canine *Borrelia burgdorferi* (*sensu lato*) infections are associated with highly elevated total IgG ELISA titers and convalescent Th2 immune responses. Curr Res Parasitol Vector Borne Dis. 2025;7:100258. 40476072 10.1016/j.crpvbd.2025.100258 PMC12138459

[10] Răileanu C, Silaghi C, Fingerle V, Margos G, Thiel C, Pfister K, et al. *Borrelia burgdorferi* Sensu Lato in questing and engorged ticks from different habitat types in Southern Germany. Microorganisms. 2021;9:1266. 34200876 10.3390/microorganisms9061266 PMC8230558

[11] Dultz R, Goldhammer M. Frühsommer-Meningoenzephalitis bei einem Hund [Tick-borne encephalitis in a dog]. Tierarztl Prax Ausg K Kleintiere Heimtiere. 2021;49:377–81. 34670313 10.1055/a-1580-8386

[12] Leschnik MW, Kirtz GC, Thalhammer JG. Tick-borne encephalitis (TBE) in dogs. Int J Med Microbiol. 2002;291:66–9. 12141763 10.1016/s1438-4221(02)80014-5

[13] Beck S, Schein E, Baldermann C, von Samson-Himmelstjerna G, Kohn B. Zeckeninfestation und Zeckenprophylaxe bei Hunden im Raum Berlin/Brandenburg–Ergebnisse einer Fragebogenstudie [Tick infestation and tick prophylaxis in dogs in the area of Berlin/Brandenburg–results of a questionnaire study]. Berl Munch Tierarztl Wochenschr. 2013;126:69–76. 23367671

[14] Schreiber C, Krücken J, Beck S, Maaz D, Pachnicke S, Krieger K, et al. Pathogens in ticks collected from dogs in Berlin/Brandenburg. Germany Parasit Vectors. 2014;7:535. 25441762 10.1186/s13071-014-0535-1 PMC4262381

[15] Selzer P, Epe C. Antiparasitics in animal health: Quo Vadis? Trends Parasitol. 2021;37:77–89. 33039282 10.1016/j.pt.2020.09.004

[16] Becskei C, Fias D, Mahabir SP, Farkas R. Efficacy of a novel oral chewable tablet containing sarolaner, moxidectin and pyrantel (Simparica Trio™) against natural flea and tick infestations on dogs presented as veterinary patients in Europe. Parasit Vectors. 2020;13:72. 32113486 10.1186/s13071-020-3946-1 PMC7049391

[17] Beugnet F, Crafford D, de Vos C, KokD Larsen D, Fourie J. Evaluation of the efficacy of monthly oral administration of afoxolaner plus milbemycin oxime (NexGard Spectra®, Merial) in the prevention of adult *Spirocerca lupi* establishment in experimentally infected dogs. Vet Parasitol. 2016;226:150–61. 27514901 10.1016/j.vetpar.2016.07.002

[18] Taenzler J, de Vos C, Roepke RKA, Heckeroth AR. Efficacy of fluralaner plus moxidectin (Bravecto® Plus spot-on solution for cats) against *Otodectes cynotis* infestations in cats. Parasit Vectors. 2018;11:595. 30449272 10.1186/s13071-018-3167-z PMC6241040

[19] Veterinärmedizinischer Informationsdienst für Arzneimittelanwendung, Toxikologie und Arzneimittelrecht (VETIDATA), https://vetidata.de/. Accessed 3 2025

[20] Petersen M, Maree R, Pretorius H, Liebenberg JE, Guerino F. Efficacy of two topical fluralaner formulations (Bravecto®; Bravecto® Plus) against Asian longhorned tick (*Haemaphysalis longicornis*) infestations of cats. Parasit Vectors. 2023;16:36. 10.1186/s13071-023-05658-8.Erratum.In:ParasitVectors.2023;16(1):74. 36703156 10.1186/s13071-023-05658-8 PMC9881378

[21] Reif KE, Bickmeier NP, Herrin BH, Dryden MW, Normile DM, Jesudoss Chelladurai JRJ, et al. Comparison of the initial and residual speed of Ixodes scapularis kill on dogs treated with a single dose of Bravecto® Chew (25 mg/kg fluralaner) or Simparica TRIO® (1.2 mg/kg sarolaner, 24 µg/kg moxidectin, 5 mg/kg pyrantel). Parasit Vectors. 2023;16:440. 38012748 10.1186/s13071-023-05946-3 PMC10683217

[22] Chiummo R, Zschiesche E, Capári B, Farkas R, Chiquet M, Rapti D, et al. Field efficacy of fluralaner (Bravecto® chewable tablets) for preventing Babesia canis infection transmitted by Dermacentor reticulatus ticks to dogs. Parasit Vectors. 2023;16:252. 37501160 10.1186/s13071-023-05820-2 PMC10373369

[23] Cavalleri D, Murphy M, Seewald W, Drake J, Nanchen S. Laboratory evaluation of the efficacy and speed of kill of lotilaner (Credelio™) against *Ixodes ricinus* ticks on cats. Parasit Vectors. 2018;11:413. 30001731 10.1186/s13071-018-2968-4 PMC6044019

[24] Geurden T, Becskei C, Grace S, Strube C, Doherty P, Liebenberg J, et al. Efficacy of a novel oral formulation of sarolaner (Simparica™) against four common tick species infesting dogs in Europe. Vet Parasitol. 2016;30:33–6. 10.1016/j.vetpar.2016.03.024 27068640

[25] Mitchell EB, McCall JW, Theodore Chester S, Larsen D. Efficacy of afoxolaner against Ixodes scapularis ticks in dogs. Vet Parasitol. 2014;201:223–5. 24685321 10.1016/j.vetpar.2014.02.015

[26] Rohdich N, Roepke RK, Zschiesche E. A randomized, blinded, controlled and multi-centered field study comparing the efficacy and safety of Bravecto (fluralaner) against Frontline (fipronil) in flea- and tick-infested dogs. Parasit Vectors. 2014;7:83. 24593931 10.1186/1756-3305-7-83 PMC3975895

[27] Taenzler J, Liebenberg J, Mienie M, Everett WR, Young DR, Vihtelic TS, et al. Efficacy of fluralaner spot-on solution against induced infestations with *Rhipicephalus sanguineus* on dogs. Parasit Vectors. 2016;9:276. 27241176 10.1186/s13071-016-1523-4 PMC4886405

[28] ESCCAP Guideline 03: Control of Ectoparasites in Dogs and Cats. European Scientific Counsel Companion Animal Parasites®. 2022. https://www.esccap.org/uploads/docs/cgqtqpf1_0720_ESCCAP_GL3__English_v19_1p.pdf. Accessed Mar 2025.

[29] CAPC guideline ticks. Companion Animal Parasite Council. 2023. https://capcvet.org/guidelines/ticks/. Accessed Mar 2025.

[30] Fisara P, Guerino F. Year-round efficacy of a single treatment of fluralaner injectable suspension (Bravecto Quantum™) against repeated infestations with *Rhipicephalus sanguineus* (sensu lato) and *Ctenocephalides felis* in dogs. Parasit Vectors. 2023;16:378. 37872632 10.1186/s13071-023-05960-5 PMC10594708

[31] Fisara P, Guerino F. Year-round efficacy of a single treatment of fluralaner injectable suspension (Bravecto QuantumTM) against repeated infestations with *Ixodes holocyclus* in dogs. Parasit Vectors. 2023;16:375. 37864235 10.1186/s13071-023-05951-6 PMC10590027

[32] Guideline on good clinical practices. VICH Topic GL9. European Medicines Agency, Committee for medicinal products for veterinary use (CVMP), 2000. https://www.ema.europa.eu/en/documents/scientific-guideline/vich-gl9-good-clinical-practices-step-7_en.pdf. Accessed Mar 2025

[33] Guideline for the testing and evaluation of the efficacy of antiparasitic substances for the treatment and prevention of tick and flea infestation in dogs and cats. European Medicines Agency, Committee for medicinal products for veterinary use (CVMP), 2016. https://www.ema.europa.eu/en/documents/scientific-guideline/guideline-testing-and-evaluation-efficacy-antiparasitic-substances-treatment-and-prevention-tick-and-flea-infestation-dogs-and-cats-revision-3_en.pdf. Accessed Mar 2025

[34] Guideline on Statistical Principles for Veterinary Clinical Trials (EMA/CVMP/EWP/81976/2010). European Medicines Agency, Committee for medicinal products for veterinary use (CVMP), 2012. https://www.ema.europa.eu/en/documents/scientific-guideline/guideline-statistical-principles-clinical-trials-veterinary-medicinal-products-pharmaceuticals_en.pdf. Accessed March 2025

[35] Marchiondo AA, Holdsworth PA, Fourie LJ, Rugg D, Hellmann K. World association for the advancement of veterinary parasitology (WAAVP) second edition: guidelines for evaluating the efficacy of parasiticides for the treatment. Prevention and control of flea and tick infestation on dogs and cats. Vet Parasitol. 2013;194:84–97. 23741753 10.1016/j.vetpar.2013.02.003

[36] Lavan R, Armstrong R, Tunceli K, Normile D. Dog owner flea/tick medication purchases in the USA. Parasites Vectors. 2018;11:581. 30400923 10.1186/s13071-018-3142-8 PMC6218982

[37] Lavan R, Tunceli K, Zhang D, Normile D, Armstrong R. Assessment of dog owner adherence to veterinarians’ flea and tick prevention recommendations in the United States using a cross-sectional survey. Parasit Vectors. 2017;10:284. 28583186 10.1186/s13071-017-2217-2 PMC5460448

[38] Lavan R, Normile D, Husain I, Singh A, Armstrong R, Heaney K. An assessment of canine ectoparasiticide administration compliance in the USA. Parasit Vectors. 2022;15:32. 35062996 10.1186/s13071-021-05134-1 PMC8780395

[39] Horizon Databook: Europe Veterinary Parasitices Market Size & Outlook. Grand View Research Inc. 2025. https://www.grandviewresearch.com/horizon/outlook/veterinary-parasiticides-market/europe. Accessed May 2025.

[40] Claxton AJ, Cramer J, Pierce C. A systematic review of the associations between dose regimens and medication compliance. Clin Ther. 2001;23:1296–310. 11558866 10.1016/s0149-2918(01)80109-0

[41] Coleman CI, Limone B, Sobieraj DM, Lee S, Roberts MS, Kaur R, et al. Dosing frequency and medication adherence in chronic disease. J Manag Care Pharm. 2012;18:527–39. 22971206 10.18553/jmcp.2012.18.7.527 PMC10438207

[42] Eisen L. Pathogen transmission in relation to duration of attachment by *Ixodes scapularis* ticks. Ticks Tick Borne Dis. 2018;9:535–42. 29398603 10.1016/j.ttbdis.2018.01.002 PMC5857464

[43] Lo Re V, Occi JL, MacGregor RR. Identifying the vector of Lyme disease. Am Fam Phys. 2004;69:1935–7. 15117014

[44] Kahl O, Janetzki-Mittmann C, Gray JS, Jonas R, Stein J, de Boer R. Risk of infection with *Borrelia burgdorferi* sensu lato for a host in relation to the duration of nymphal I*xodes ricinus *feeding and the method of tick removal. Zentralbl Bakteriol. 1998;287:41–52. 9532263 10.1016/s0934-8840(98)80142-4

[45] Fourie JJ, Evans A, Labuschagne M, Crafford D, Madder M, Pollmeier M, et al. Transmission of Anaplasma phagocytophilum (Foggie, 1949) by Ixodes ricinus (Linnaeus, 1758) ticks feeding on dogs and artificial membranes. Parasit Vectors. 2019;12:136. 30909972 10.1186/s13071-019-3396-9 PMC6434881

